# Contrasting the Material Chemistry of Cu_2_ZnSnSe_4_ and Cu_2_ZnSnS_(4–_ *_x_*_)_Se*_x_*

**DOI:** 10.1002/advs.201500320

**Published:** 2016-02-02

**Authors:** Jeffery A. Aguiar, Maulik Patel, Toshihiro Aoki, Sarah Wozny, Mowafak Al‐Jassim

**Affiliations:** ^1^ National Renewable Energy LaboratoryGoldenCO80401USA; ^2^ Department of Materials Science and EngineeringUniversity of Tennessee‐KnoxvilleKnoxvilleTN37996USA; ^3^ LeRoy Center for Solid State ScienceArizona State UniversityTempeAZ85281USA; ^4^ Advanced Materials Research InstituteUniversity of New OrleansNew OrleansLA70148USA

**Keywords:** CZTS, photovoltaics, solar cells, STEM, thin‐films

## Abstract

**Earth‐abundant sustainable inorganic thin‐film solar cells,** independent of precious elements, pivot on a marginal material phase space targeting specific compounds. Advanced materials characterization efforts are necessary to expose the roles of microstructure, chemistry, and interfaces. Herein, the earth‐abundant solar cell device, Cu_2_ZnSnS_(4–_ *_x_*_)_Se*_x_*, is reported, which shows a high abundance of secondary phases compared to similarly grown Cu_2_ZnSnSe_4_.

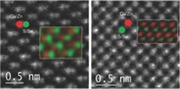

Demand for new power plants relying on solar technology keeps rising as investments increase, and declining production costs are mounting a competitive edge over other energy hosts. In order to further support solar technology as a competitive and secure energy resource and reach grid parity with fossil fuels, the elements of the cell must be chosen to reflect current and future mineral supplies, as well as address environmental sustainability concerns. Avoiding the use of scarce, precious, and potentially toxic minerals, such as gallium, indium, and cadmium should be considered.^1^ Given the natural abundance of precursor materials and low toxicity of the final inorganic phase, Cu_2_ZnSnS_4_ (CZTS) based solar cells are potential candidates for future sustainable energy production. However, additional materials‐level research to support the use of these earth‐abundant materials within a solar cell is necessary to overcome current technological barriers. These barriers are mostly material derived and include a constrained material phase‐space where CZTS is a line compound within that framework, and there is an expectation to share the same tailored band‐structures and device efficiencies (>15%) as other competitive photovoltaics.^2^, ^3^, ^4^ The technical approach to advance the field of earth‐abundant photovoltaics, therefore, hinges on the incorporation of several fundamental material insights and studies into the atomic structure, chemistry, and generated defects across multiple length scales and growth techniques.^5^, ^6^, ^7^

In light of the inherent challenges described with studying alternative photovoltaics absorber materials such as I_2_‐II‐IV‐VI_4_ CZTS, CZTSe, and CZT(S,Se) semiconductors, these same materials have attracted broad interest as a result of the highly variable and complicated relationships between atomic ordering, chemistry, and device properties.^8^, ^9^, ^10^, ^11^ Common to these I_2_‐II‐IV‐VI_4_ semiconductors is the inherent variability in material chemistry, specifically the formation of nonstoichiometric sulphide, selenide, and sulphoselenide secondary phases. The difficulty associated with this class of semiconductors is the lack of thermodynamic stability of a line‐compound and losses of Sn and S species during annealing due to low vapor pressures. The effects are further propagated through thermal treatments which result in the decomposition of the quaternary chalcogenide due to the formation of secondary phases such as Cu_2_SnS_3_, Cu_2_SnSe_3_, Cu_2_Se_3_, CuSe_2_, ZnS, and others,^3^, ^12^ which ultimately hinders the final device photovoltaic performances. Despite these challenges, I_2_‐II‐IV‐VI_4_ thin‐films devices with efficiency higher than 10% have been achieved by vacuum^13^ and nonvacuum^14^ based techniques. These advanced studies however lack the direct point–point correlation between measured material chemistry, resultant electronic structure, and device performance. Reports on the atomic arrangements and chemical profiles associated with these semiconductors therefore serve as vital material studies.^9^, ^15^, ^16^, ^17^ In solar cell technology, explaining differences affecting the conduction and valence band energies, especially in the vicinity of the space charge region, has important consequences on derived performance and ultimate directions for device processing.^6^, ^7^, ^18^, ^19^

In this study, we apply analytical scanning transmission electron microscopy (STEM) and spectroscopy to report on differences in grain**‐**to**‐**grain specific chemistry, atomic structure, and electronic structure between CZTSe and CZTSSe. Here, we specifically show a high percentage of nonstoichiometric grain‐to‐grain compound formation, in both CZTSSe and CZTSe thin‐film. A lack of uniformity further presents itself as not only differences in material chemistry, but in the measured valence electronic structure using electron energy loss spectroscopy (EELS). We presume that nonstoichiometric compound formation leads to a lower than expected device efficiency due to resolved stoichiometric differences that give rise to a spatially varying electrostatic potential. In turn, these deviations reduce the carrier lifetime, carrier separation, collection efficiency, and hence the overall device performance. Overall, the results shared in this study represent a point of intersection with the current literature and provide further insights into the phase stability, resultant material chemistry, and related electronic structure of CZTS thin‐film solar cells.

CZTSe and CZTSSe devices were grown using thermal co‐evaporation, as previously reported in the literature.^20^ Each sample was constituted of a stack of metallic and semiconducting layers consisting of a soda‐lime glass substrate, a sputtered molybdenum (Mo) back contact, an e‐beam evaporated NaF precursor (150 Å), a co‐evaporated CZTSe or CZTSSe absorber layer (1–3 μm), a CdS buffer layer (500 Å), a ZnO bilayer (0.2 μm), an MgF_2_ antireflection coating layer, and Ni/Al grids. Further details on the chalcogenide deposition and annealing processes and final device structure can be found in the literature.^20^ Both CZTSe and CZTSSe final films composition had target average Cu/Zn ratio of 2 terminating Zn‐poor, consistent with a Cu rich absorber material. Each of the devices photovoltaic performances were evaluated with current–density versus voltage (*JV*) plots. *JV* curves were collected under simulated AM 1.5G illumination (light irradiation of 100 mW cm^−2^), and device performances are reported in **Table** **1**. A 5.6% device conversion efficiency was measured for CZTSSe and further confirmed using quantum efficiency measurements. The open circuit voltage (*V*_oc_ = 0.316) was lower than expected based on band gap expansion predicted for the amount of S present.^21^ Similarly grown CZTSe demonstrated a final power conversion efficiency of 9%. The average composition of the two devices is Cu_1.91_Zn_0.99_Sn_1.01_Se_4.09_ and Cu_1.92_Zn_0.99_Sn_1.02_S_3.15_Se_0.85_. Overall, comparing the *JV* measurements between the grown CZTSe and CZTSSe leads to nearly an order of magnitude difference.

**Table 1:** Device Characteristics of CZTSe and CZTSSe

	CZTSe	CZTSSe
*V*_oc_ [mV]	0.369	0.316
*J*_sc_ [mA cm^−2^]	35.62	29.21
FF [%]	68.42	60.13
*η* (%)	8.983%	5.552%

CZTSe and CZTSSe devices were first studied with aberration corrected STEM imaging. **Figure** **1** is a comparison of the material grain‐to‐grain chemistry between CZTSe and CZTSSe devices. Starting from the top of the image, Figure 1a is a STEM high‐angle annular dark field (HAADF) image of the CZTSe device cross‐section. This image reveals the device structure, consisting of the transparent conducting ZnO window layer from the top of the device, followed by the n‐type junction layer. Below the CdS buffer is the CZTSe absorber layer, followed by the molybdenum back contact electrode. The same solar cell structure and layering is similarly shown in the STEM HAADF image, Figure 1b, for the CZTSSe device.

**Figure 1:**
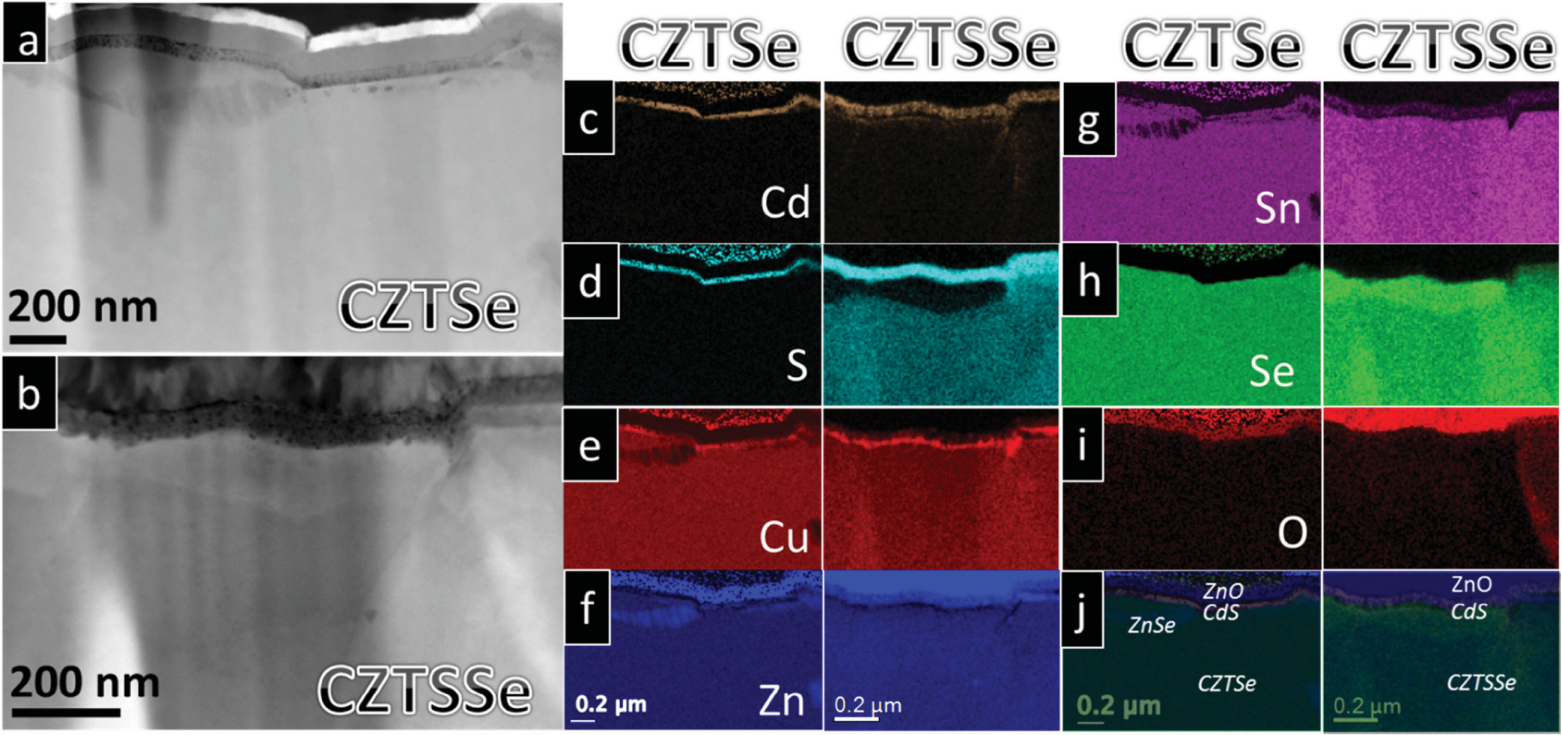
A comparison between CZTSe and CZTSSe samples was performed using analytical microscopy. a) Starting at the top of the image, STEM HAADF structural imaging reveals uniform yet rough layering of ZnO and CdS followed by CZTSe and the back contact material Mo. b) The same solar cell structure and morphology is evident in the CZTSSe cell. For each element c) Cd, d) S, e) Cu, f) Zn, g) Sn, h) Se, and i) O, we resolve and compare chemical morphology between CZTSe and CZTSSe. Please note above the ZnO layer, there is vacuum, and therefore EDS signal noise, these counts are not representative of elements over this region.

Over the same area scanned, to expose differences in bonding, periodicity, mass, and chemistry at these grain boundaries, STEM‐based chemical imaging was performed combining both EELS and energy dispersive X‐ray spectroscopy (EDS). For each of the elements, we compare and contrast the chemical morphology between the CZTSe and CZTSSe devices for Cd **(**Figure 1c), S (Figure 1d), Cu (Figure 1e), Zn (Figure 1f), Sn (Figure 1g), Se (Figure 1h), and O (Figure 1i).

The material chemistry similarities between the two devices include the presence of secondary phases. In both cases, a secondary phase made of zinc and selenium is visible at the absorber surface based of the Zn **(**Figure 1f) and Se **(**Figure 1h) maps as expected from the Zn‐rich termination during growth. This layer is not uniform over the length of the heterojunction with CdS in either case. In those same regions of zinc selenide, we also note that tin is depleted (Figure 1g) because it shares the same cation site as zinc in both CZTSe and CZTSSe. This same behavior has also been noted in several reports in the literature and is not unique to rapid quenched thin‐film compounds.^4^, ^6^, ^7^, ^19^ CZTS also shows similar secondary phases in the literature.^22^, ^23^

For thin‐film solar cells, the presence of secondary phases, especially at and in vicinity of the junction region presents a series of implications on the probability that minority carriers (electrons in p‐type CZTSe and CZTSSe) can diffuse to the edge of the depletion region. This, in turn, reduces carrier separation efficiency. On the other hand, at the holes from the n‐type semiconductor and those photogenerated within the absorber layer can become “trapped” at these potential wells, reducing the overall carrier efficiency due to the presence of antibonding with the valence band maximum. The presence of a Zn‐rich layer is estimated as ≈10 nm, where the size of Wannier–Mott excitons in semiconductors is on the order of a few nanometers.^24^ Comparing the relative sizes of the composition inhomogeneity suggests that the recombination of electrons and holes is dominant within both CZTSe and CZTSSe.

Despite presumably identical growth conditions between CZTSe and CZTSSe, there are discerning differences in material chemistry. Differences between CZTSe and CZTSSe include Cd enrichment at grain boundaries in CZTSSe, as well as non­uniform sulfur, copper, zinc, tin, selenium, and oxygen stoichiometry. Sulfur content presumably from the chemical bath deposition of CdS migrates into the grain interiors of CZTSe, but at a lower atomic percent compared to CZTSSe. The formation of Cd enrichment resolved at grain boundaries in Figure 1c for CZTSSe is significant. We speculate the presence of CdS at the grain interiors is due to a lack of grain to grain uniformity. Further, we hypothesize that this is leading to interior grain diffusion, where Cd^2+^ is presumably acting as a grain boundary stabilizer. Under this presumption, CdS would readily diffuse into the CZTSSe due to the presence of defect content along the grain boundaries, forming extended vertical heterojunctions. Beyond Cd and S, elements such as Cu (Figure 1e), Zn (Figure 1f), Sn (Figure 1g), Se (Figure 1h), and O (Figure 1j) also share the differences from grain to grain. Based on the measurable variances between the two devices, we presume the lower than expected device performance is related to the lack of consistent stoichiometry. These results further suggest a closer atomic structure comparison between CZTSe and CZTSSe.

CZTSe and CZTSSe were further studied to compare and resolve any differences in atomic ordering between the two devices. The comparison to CZTSe starts with a high‐resolution micrograph shown in **Figure** **2**a of CZTSe, where a specific grain was oriented to the [001] beam direction resolved using selected area electron diffraction (SAED), shown in Figure 2b. To resolve any differences in lattice ordering along the [100]_CZTSe_ beam direction, a selected region of the sample shown in Figure 2c was analyzed using column–column STEM‐based EELS. Figure 2d is the atomic column‐by‐column CZTSe chemistry taken for Cu‐*L*, Zn‐*L*, Sn‐*L*, and Se‐*K* using STEM‐EELS. Based on these collected images, we resolve no clear differences outside of the expected lattice between the Cu/Zn and Se atomic columns. We must however note that the Se column may also contain a low concentration of sulfur based on the chemical image presented in Figure 1d due to CdS diffusion but to a very minimal amount compared to the CZTSSe cell.

**Figure 2:**
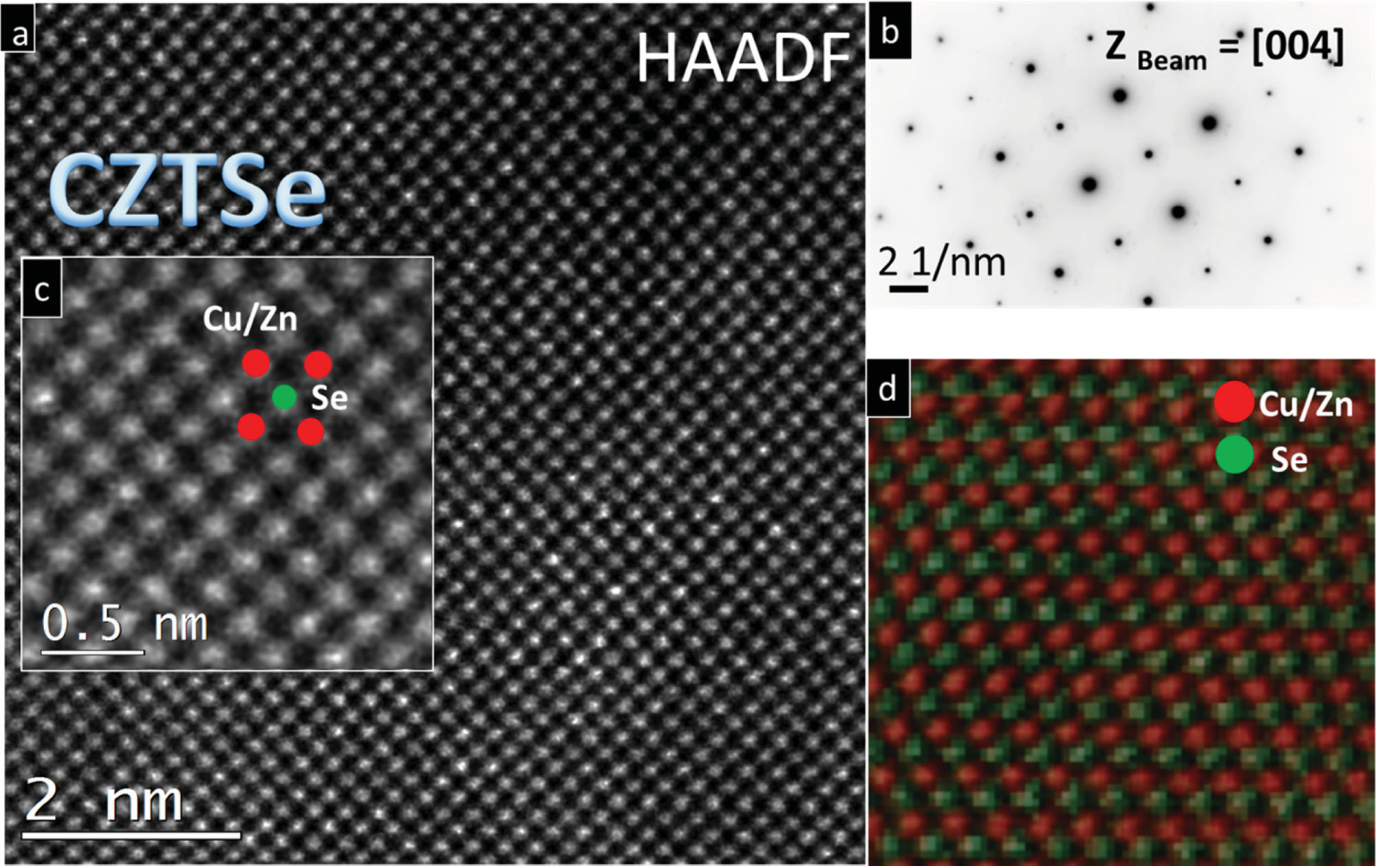
a) An atomic contrast STEM image of CZTSe taken along the [100]_CIGS_ beam direction, b) confirmed using selected area electron diffraction. c) Closer look at the atomic contrast image reveals d) alternating Cu/Zn and S columns using STEM EELS column–column chemistry.

Similarly, we performed high‐resolution STEM imaging to discern differences in atomic contrast for CZTSSe. We oriented CZTSSe to the [111]_CZTSSe_ zone axis shown in **Figure** **3**a and confirmed our orientation using SAED in Figure 3b. To distinguish differences in atomic contrast, a closer look at the simultaneous HAADF and annular bright field (ABF) imaging shown in Figure 3c,d, respectively, reveals the Cu/Sn and S/Se atomic columns. A closer look at the HAADF (Figure 3c) and ABF (Figure 3d) image reveals not only the differences in intensity associated with Cu/Zn and S/Se columns, but resolves differences in scattering potential (i.e., ionic size). To confirm differences in atomic column intensity and ionic size resolved in the HAADF STEM images, Figure 3e is the acquired STEM‐EELS column–column map for CZTSSe. The expected atomic lattice is confirmed based on the Cu/Zn and S/Se column–column chemistry. Based on both the structural and chemical imaging, we do not observe atomic scattering differences due to lattice ordering, but rather cannot distinguish. STEM thereby cannot definitively disassociate an ordered structure for either CZTSe or CZTSSe.^9^, ^25^

**Figure 3:**
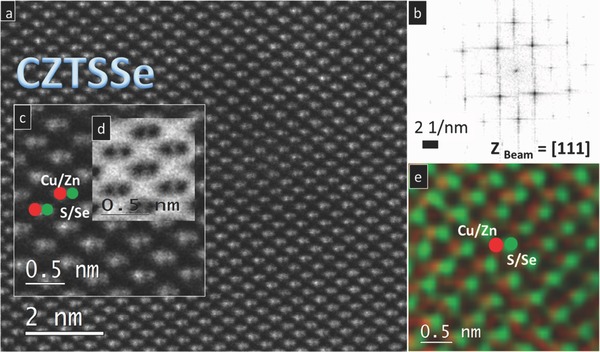
a) High‐resolution STEM imaging along the [111]_CIGS_ zone axis and b) confirmed using selected area electron diffraction. c) A closer look using simultaneous annular dark and d) bright field imaging clearly identifies column‐by‐column intensity. e) Alternating Cu/Zn and S/Se columns are confirmed using STEM EELS column–column chemistry.

Aware of the differences in grain‐to‐grain stoichiometry in CZTSSe, we turn our attention to report on the electronic structure associated with the device using valence EELS. Across a series of grains and boundaries, shown in **Figure** **4**a, we performed STEM EELS linescans. Figure 4b is a horizontal linescan across multiple grains and boundaries. This linescan progressively changes across the CZTSSe grains. Certainly the differences in stoichiometry are giving rise to differences. Specifically, we observe the presence of the 0.7 eV energy‐loss feature and the shape of the 1.25 eV peak from grain to grain. The CZTSSe feature at roughly 0.7 eV is certainly below the expected band gap for this material (1.0–1.5 eV). This spectral profile therefore identifies the presence of a mid‐gap state between the conduction band minimum and valence band maximum.^26^ In terms of the overall device, valence EELS specifically confirms the presence of a spatially varying electrostatic potential from grain to grain that causes differences in the electronic transitions between the presumed donor and acceptor levels. This is a series of important results that consequentially alter the final electronic and device properties due to differences in band‐gap alignment between the CdS junction and the absorber layer.

**Figure 4:**
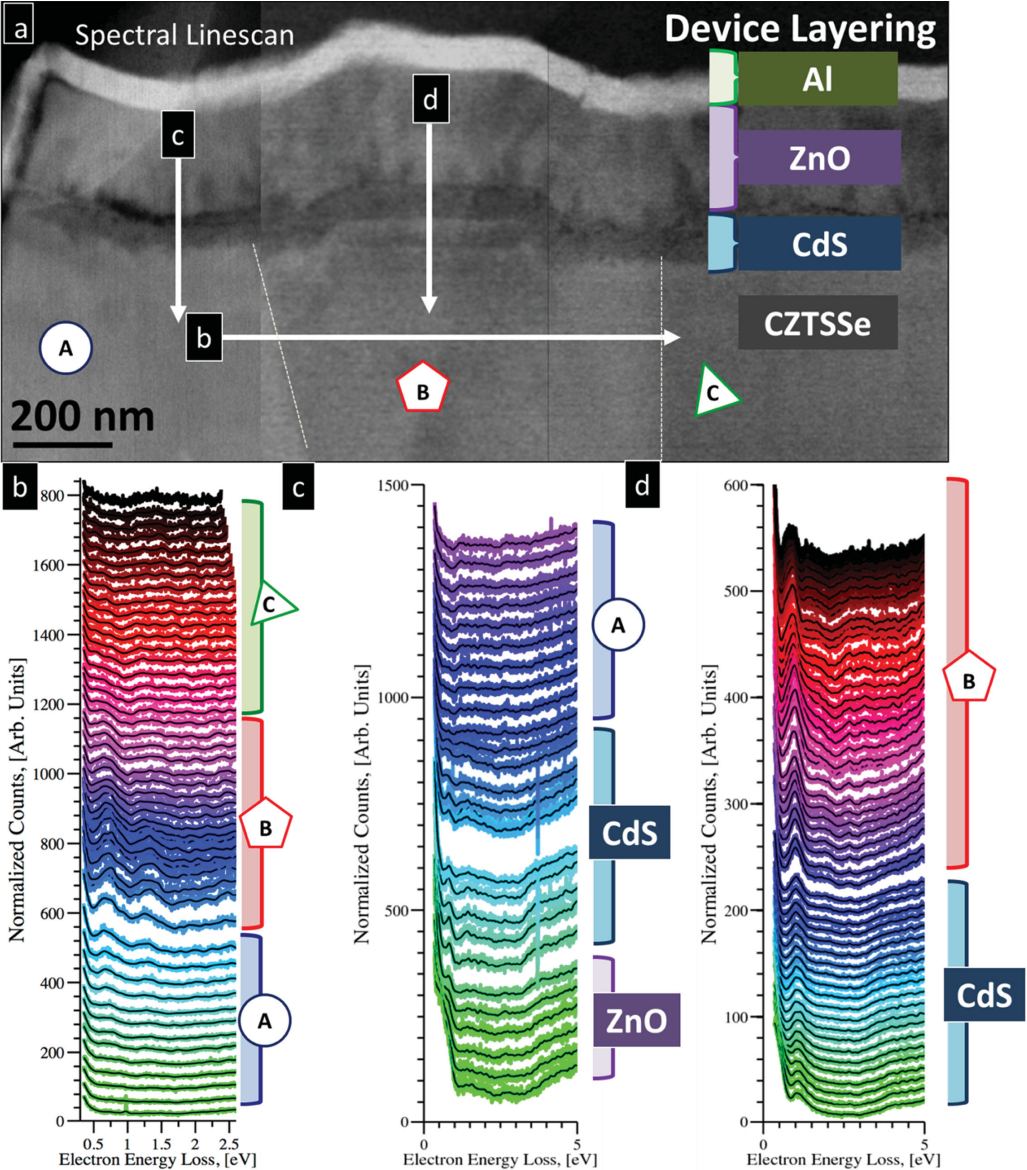
a) Point resolved STEM‐EELS spectroscopy was along and perpendicular to the CZTSSe cross‐section device. b) We resolve spectral differences related to grain chemistry by progressively reporting differences from grain to grain. c–d) Similarly, we performed EELS linescans over next two CZTSSe grains traversing ZnO/CdS/CZTSSe layers reported as offset scatter plots. The valence energy loss spectra reported are at each point separated by 1 nm. Each of the symbols identifies different CZTSSe grains, where the dashed white lines demark grain boundaries between each other.

To follow, we performed VEELS over length of the CZTSSe device cross‐section. We performed three separate vertical VEELS linescans starting from the ZnO layer, continuing to CdS buffer layer and ending in CZTSSe chalcogenide. Each of these linescans reports on the trending differences in the near‐IR to valence region associated with the material. In the first vertical linescan, Figure 4c resolves the near valence electronic structure, where each point is separated by 1 nm and displayed as an offset scatter plot. Figure 4d is a similarly performed linescan over the CdS and CZTSSe junction, ending in a separate grain, respectively.

Unlike the grain‐to‐grain linescan, comparing these three separate linescans, Figure 4c,d reveals a trending difference to a higher band‐gap offset as function of probe position. In Figure 4c the CZTSSe grain probed is consistent with a significant amount of oxygen, as well as the depletion of copper and selenium. The grain chemistry is vastly different than the other two grains. The second STEM‐VEELS linescan shown in Figure 4d resolves a clear band gap offset at 1.18 eV with no lower optical features, dissimilar to the previous two linescans. These points clearly identify differences in band‐offsets that depend on the CZTSSe grain chemistry, especially in the vicinity of the CdS heterojunction. The presence of nonstoichiometric CZTSSe not only showcases the departure from uniform device chemistry but highlights the crucial role it plays in the performance of the device, in particular its effect on the valence electronic structure.

From the detailed characterization in this study, we report that the lack of stoichiometry in CZTSSe compared to CZTSe leads to altered band‐gap offsets and the presence of undesired electro‐optical features. We speculate that the lack of stoichiometry in CZTSSe drastically affects the performance of the final photovoltaic device. In the present case, we find the buildup of Cd decorates grain boundaries in CZTSSe, where the lack of stoichiometry can significantly accelerate the accumulation of elements at open‐surfaces and act as a stabilizing agent. This is beyond the void structure observed in previous literature reports and extends well below the junction region.^27^ More importantly, we have found in this study that this particular growth process and presumably co‐evaporation of SnS, lead to a variation from the expected grain morphology and device performance. We have demonstrated that the importance of grain chemistry can play a detrimental role on the measured device properties and ultimate performance of the solar cell. These points further reiterate the need for in situ studies to track and control the material chemistry of this otherwise complex quaternary polycrystalline chalcogenide material during growth.

A systematic experimental investigation of the structure, chemistry, and electronic structure of polycrystalline CZTSe and CZTSSe was investigated. The goal was to report on the complex relationship of material chemistry and its effects for a set of quaternary polycrystalline inorganic thin‐film photo­voltaics by high‐resolution analytical electron microscopy.

Our results demonstrate that polycrystalline CZTSSe shows higher amount of secondary phases and nonstoichiometry compared to similarly grown CZTSe. Detailed materials characterization enabled us to profile both CZTSe and CZTSSe following growth, accounting for changes in material chemistry and electronic structure. We find that grain interiors and their interfaces are centers for differences in secondary compound formation. The grain interiors lead to presumably destabilized interface formation energies that were compensated by the presence of cadmium cations. We suggest that the lack of consistent stoichiometry in CZTSSe destabilizes cell and leads to a lower than expected performance of the cell.

In conclusion, our results support the idea that the junction and lack of stoichiometry in polycrystalline CZTSe‐related solar cells is otherwise related to the final device performance of the cell. This study indicates that a heightened attention to resultant grain structure and chemistry is warranted to tailor the responses and efficiency of future sustainable earth‐abundant photovoltaics.

## Experimental Section

*Microscopy Sample Preparation*: Electron transparent samples were obtained utilizing the standard focus ion beam (FIB) lift‐out technique for the same areas. A final thinning was performed using a 5 kV accelerating voltage and a beam current of 12 pA to remove material redeposited during the TEM FIB lift‐out process and reduce the damage from the initial 30 kV ion milling, followed by low energy cleaning at 600 eV and 170 °C, for ±10°, in a Fischione nanomill FIB instrument. Care was taken to minimize the ion beam interaction with the face of the sample throughout the TEM sample preparation. The initial standard lift‐out was done using a typical sample size of about 10 μm × 30 μm and a thickness of ≈2 μm prior to further thinning to electron transparency. The electron transparent region observed in TEM was only about 8 μm × 5 μm.

*Analytical Microscopy*: Analytical transmission electron microscopy was performed on the probe‐corrected JEOL ARM 200F, FEI ChemiSTEM, and FEI Titan S. High‐resolution atomic contrast imaging was performed on the JEOL ARM operated in STEM mode at 200 kV with 20 mrad semi‐convergence angle and equipped with a Gatan Enfinium ER electron energy loss image filter, high solid angle 50 mm^2^ X‐ray detector, and the latest precession electron diffraction system from AppFive located at the LeRoy Eyring Center for Solid State Science at Arizona State University. The FEI ChemiSTEM operated at 200 kV located at Sandia National laboratory performed electron dispersive X‐ray spectral (EDS) chemical imaging using four simultaneous solid‐state EDS detectors to acquire the Cu‐*L*, Sn‐*K*, Zn‐*L*, Se‐*K*, Zn*‐K*, Zn*‐L*, Cd‐*K*, and S‐*L* edges with the best achievable spatial and energy resolution for the microscope. The acquisition time to resolve the EDS was performed over a series of consecutive subsecond exposures over a period of 1 h. The EDS data was then processed using hyperspectral component image analysis, where the significant components are matched to the individual phases within the material.^28^ In brief, the composite hyperspectral images resolve the individual major component spectra, and resolve each of those as a composite image. The probe‐corrected FEI Titan S located at Oak Ridge National Laboratory was also utilized to perform complementary high‐resolution imaging at 300 kV operated in STEM mode with a 22.4 mrad convergence angle.
